# Breast Reduction in Behçet's Disease: A Case Report

**DOI:** 10.7759/cureus.45031

**Published:** 2023-09-11

**Authors:** Safvet Ors

**Affiliations:** 1 Plastic and Reconstructive Surgery, SO-EP Aesthetic and Plastic Surgery Clinic, Kayseri, TUR

**Keywords:** behçet's disease, inferior pedicle technique, vasculitis, colchicine, reduction mammoplasty

## Abstract

Behçet's disease (BD) is an inflammatory systemic vasculitis manifested by uveitis and oral and genital aphthae. There have been no reported cases in the literature about reduction mammoplasty or large flap surgeries in BD. A 55-year-old female with a 16-year history of BD was presented to our clinic for reduction mammoplasty. The patient was taking colchicine and had records of uveitis and oral and genital aphthae. Colchicine treatment was stopped one week before surgery and restarted one week later. Reduction mammoplasty was performed with the inferior pedicle technique. On the first day after surgery, mild hyperemia and induration were observed around the incision line, worsening over the next 48-72 hours with the appearance of serous discharge at the vertical suture line and inverted T incision. Minimal wound dehiscence developed on the seventh day, and a small superficial necrosis developed at the lower part of the vertical suture on the 15th day. Between days 30 and 45, the necrotic tissue sloughed off, and the wound closed with epithelialization. In conclusion, patients with BD may experience prolonged inflammation, small skin necroses around the incision line, and delayed wound healing post surgery, although acceptable scar maturation can eventually be achieved.

## Introduction

Behçet's disease (BD) is an inflammatory systemic vasculitis manifested by uveitis and oral and genital aphthae. BD can also affect the vascular system, joints, and gastrointestinal and central nervous systems [1-4]. Various surgical procedures such as abdominal surgery, cardiovascular surgery, breast cancer surgery, rhinoplasty, and graft transfer were reported in patients with BD [1-7]. In some of these reports, it was reported that wound healing was impaired and improved with the use of colchicine [6]. We specify that no case reported delayed healing after reduction mammoplasty in BD.

## Case presentation

**Figure 1:**
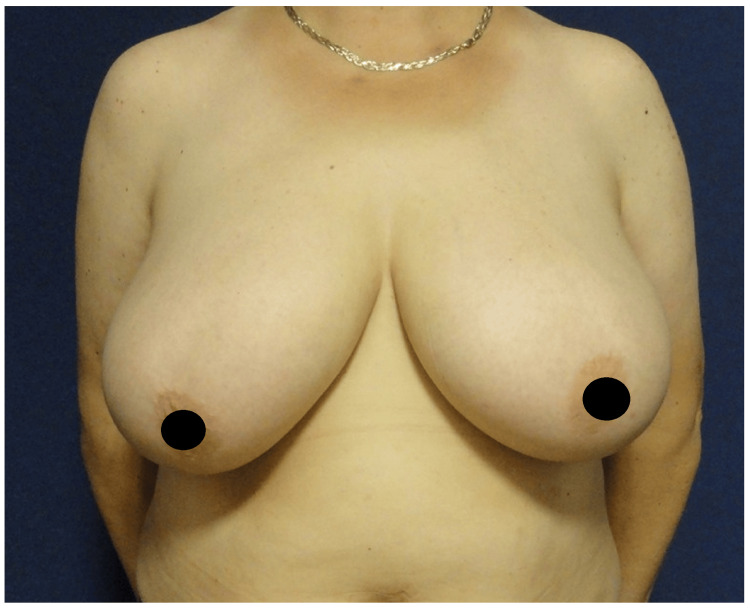
Breast reduction preoperative view

A 55-year-old female with a 16-year history of BD was presented to our clinic for reduction mammoplasty in 2016 (Figure 1).

**Figure 2:**
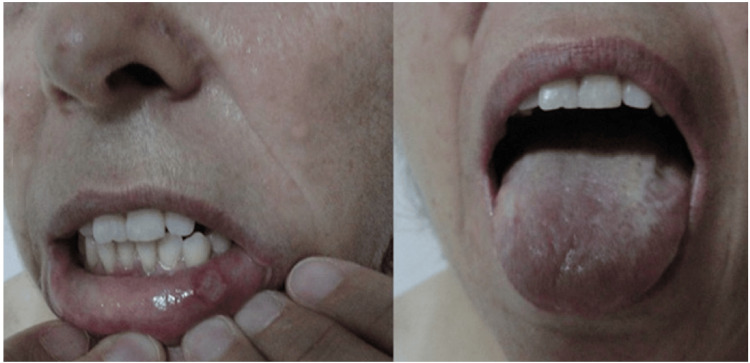
Active aphthae were observed on the oral mucosa and tongue of the female patient with Behçet's disease

The patient was taking colchicine and had records of uveitis and oral and genital aphthae (Figure 2). The patient did not have any disease that would adversely affect wound healing, such as diabetes and morbid obesity.

**Figure 3:**
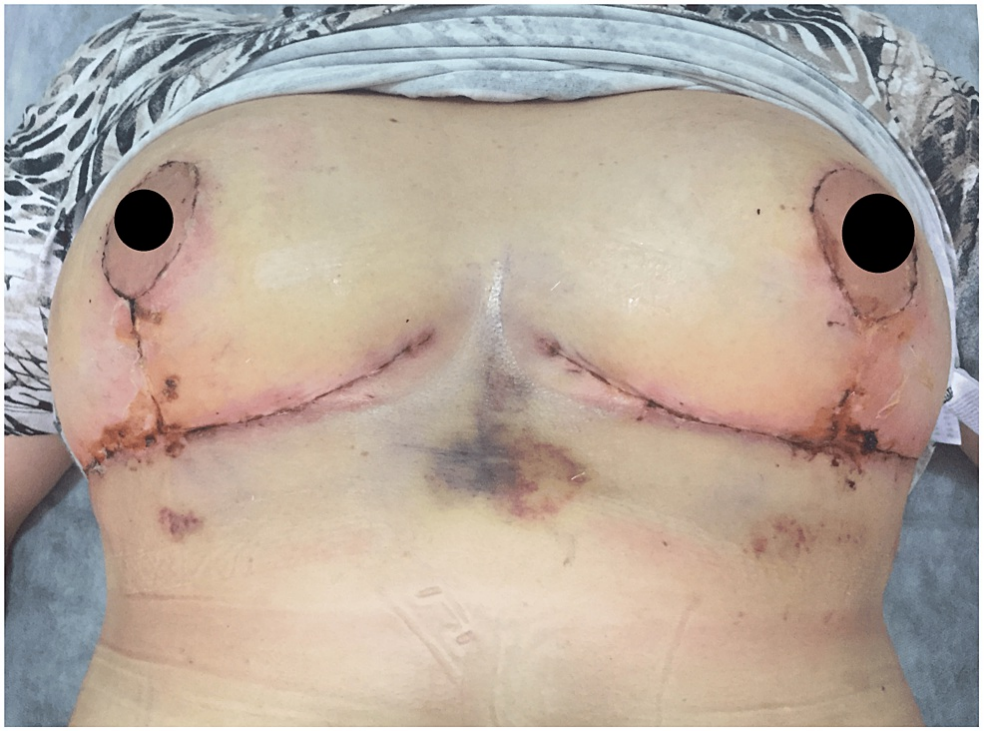
Inflammation, edema, and serous discharge observed in and around the incision line

Colchicine treatment was discontinued one week before surgery based on the immunologist's recommendation. Colchicine was restarted early, as postoperative inflammation increased a lot. The nipple-areola complex (NAC) was planned to be reduced from 27 cm to 21 cm on the left and from 30 cm to 21 cm on the right using wise pattern with inferior pedicle technique. During surgery, vascular blood supply was observed to be very sufficient up to the most distal part of the flaps. The flaps were closed with 3/0 polydioxanone (PDS) and 4/0 polyglecaprone (Monocryl, Ethicon, Inc., Raritan, NJ) sutures. The patient was closely monitored postoperatively for cardiac, renal, and pulmonary complications. For the first three days after surgery, the patient was administered 500 mL of 10% Rheomacrodex intravenously and 100 mg of salicylic acid orally. On the first day after surgery, mild hyperemia and induration were observed around the incision line, worsening over the next 48-72 hours with the appearance of serous discharge at the vertical suture line and inverted T incision (Figure 3).

**Figure 4:**
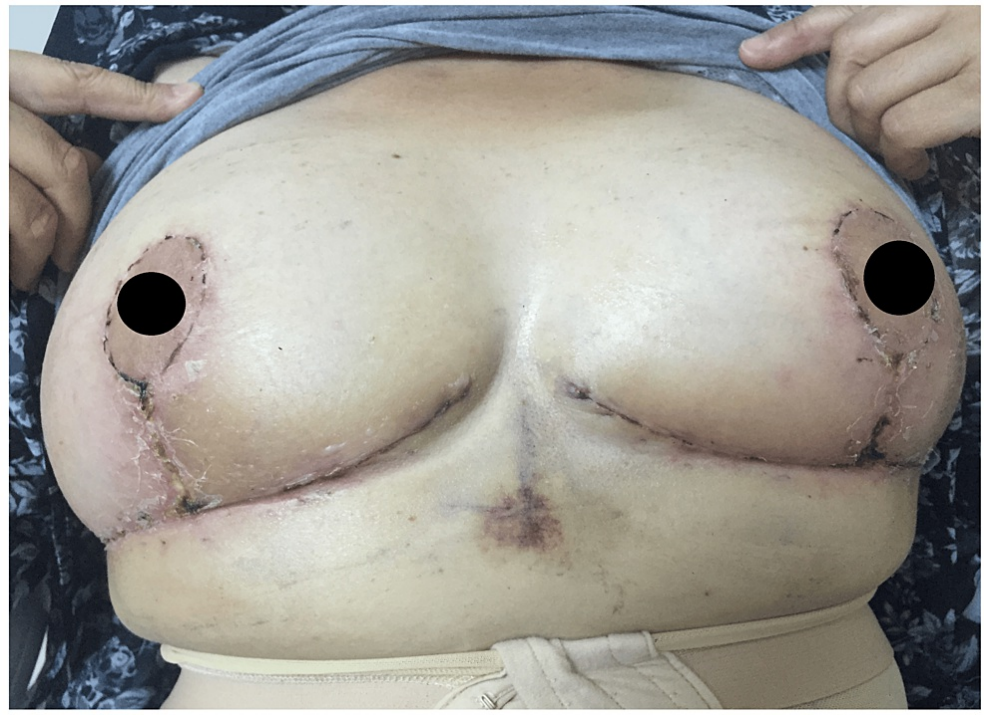
Approximately 15 days after surgery, minimal wound dehiscence and small superficial necrotic areas were observed at the lower part of the vertical suture line

The patient was discharged three days after the operation, and daily controls were made. Minimal wound dehiscence developed on the seventh day, and a small superficial necrosis with a diameter of 1 cm developed at the most distal part of the vertical suture on the 15th day (Figure 4).

**Figure 5:**
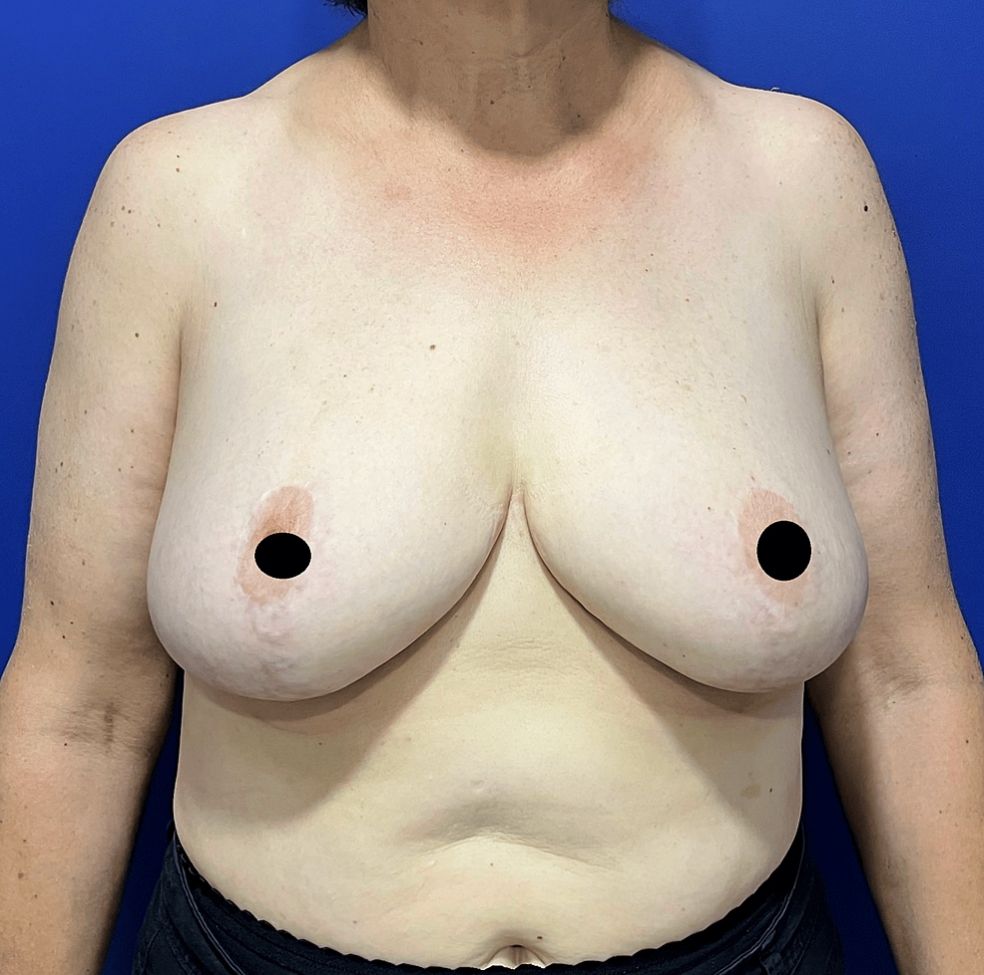
The appearance of the wound after the sloughing of necrotic tissue and the closure of the wound through epithelialization on postoperative year 8

These necroses were not debrided but were expected to heal with epithelialization. Between days 30 and 45, the necrotic tissue sloughed off, and the wound closed with epithelialization (Figure 5).

No necrosis was observed in the NAC, and the patient ultimately recovered with an acceptable scar. The patient underwent antiscar treatment with silicone cream. The patient was followed up for eight years postoperatively, during which time scar maturation and wound healing were quite acceptable (Figure 5).

## Discussion

Wound healing begins with platelet adhesion and continues with inflammation. Tissue blood supply can be disrupted due to leukocytic vasculitis, and tissue necrosis may occur even with minor traumas in BD [2,5,8,9]. In Behçet's disease, the period of inflammation, especially in the skin, is prolonged as a result of surgical trauma [8,9]. Purulent discharge and partial wound dehiscence seen in the incision line in Behçet's patients are due to the prolongation of the inflammation period. However, Behçet's disease does not directly impair wound healing [9]. The disease can affect small vessels throughout the body, potentially causing a decrease in blood flow and blood supply problems in surgical flaps due to vasculitis. There were no studies reported investigating the recovery process in BD after surgeries in which large tissue flaps are used, such as reduction mammoplasty. When considering plastic surgery in BD, physicians must keep two important factors in mind: wound healing and the impact of medications on the healing process. In BD, inflammation is typically prolonged, and wound dehiscence and serous discharges may be seen. There have been various studies of wound healing in BD to date. In one study, involving punch biopsies with a diameter of 4 mm in patients with BD, erythematous areas were observed around the wound within 24 hours, followed by inflammatory changes and induration within 48 hours, along with purulent and serous discharges. Complete wound healing typically occurs within 10-15 days, despite the increased inflammatory response [8]. According to this study, biopsy-induced trauma may cause increased inflammation in BD, but wound healing is not altered [8]. In the present case, similar results were observed, despite the much larger surgical area. The small area of necrosis observed on the incision line was likely related to the width of the surgical field and the elevated flap-shaped tissue. In another study, wound dehiscence at the incision line was observed in a patient with Behçet's disease who was operated for hydrocele. It has been reported that the incision of this patient was reopened after the revision but improved after he started using low-dose colchicine [6].

Colchicine mainly binds to intracellular tubulin and inhibits microtubule polymerization. They are responsible for the shape of the cell, the transfer of intracellular substances, the release of cytokines and chemokines, cell migration, the regulation of the activity of ion channels, and cell division processes. Colchicine limits mitochondrial activity and stops mitosis in metaphase with microtubule inhibition. It inhibits the chemotaxis of inflammatory cells, endosome, and exosome transport. It also inhibits caspase-1 activation and interleukin (IL)-1 and IL-8 release by preventing inflammasome activation. It also reduces the effect of monocytes and neutrophils by decreasing the expression of E-selectin, which is an important adhesion molecule. It also reduces the production of free oxygen radicals by neutrophils and suppresses inflammation in many stages. Researchers reported exaggerated inflammation in patients with BD that lasted longer than in healthy patients [8,9].

Experimental studies have shown that the use of colchicine in random pattern flaps significantly reduces the area of ​​necrosis in the flap [10]. In this study, it was reported that interleukin-6 and tumor necrosis factor-α levels decreased and N-methyl-D-aspartate (NMDA) receptors increased in flaps in the colchicine group. Since breast reduction surgery is also a type of flap surgery, we observed that the necrosis did not expand as a result of the use of low-dose colchicine in our patient. Salai et al. showed that low-dose colchicine did not affect cell proliferation but inhibited at high dose [11]. The use of topical colchicine significantly prevents fibrosis in rats, resulting in better wound healing [12]. Colchicine prevents ischemia-reperfusion injury in cardiac surgery and reduces the recurrence of intra-abdominal adhesions and pericarditis [13-15]. According to Peacock, colchicine prevents hypertrophic scar formation by stimulating tissue collagenase activity and correcting the abnormal balance between collagen synthesis and collagenolysis [16]. As a result, low-dose colchicine, which is used after cardiac surgery, joint surgery, and other operations, accelerates wound healing in Behçet's disease patients.

Colchicine was discontinued one week before surgery with the advice of an immunologist. After the operation, low-dose colchicine was restarted due to the high inflammation. Cyclophosphamide, cyclosporine A, corticosteroids, and colchicine are widely used in the medical treatment of BD, even after surgery [5-7]. Colchicine is perhaps the drug that affects wound healing the least compared to other agents. We believe that although low-dose colchicine partially reduces the inflammatory response, it will not impair wound healing. However, high doses of colchicine may impair wound healing. Nevertheless, several studies have found that colchicine can positively affect the healing process by reducing inflammation and may even prevent hypertrophic scar formation [13-18]. In the present study, colchicine treatment was suspended one week before surgery and restarted one week after surgery. The important thing here is the dose of colchicine. If possible, low-dose use would be appropriate after surgery. The number of studies on colchicine is limited [6,10]. We had to start the colchicine again as the inflammation increased too much after operation. We hypothesize that the administration of low-dose colchicine in the early postoperative period may reduce inflammation, limits necrosis, and accelerates healing. We opted for this approach in our patient, despite the limited availability of studies on the topic, and based on the results, we can recommend that colchicine be continued postoperatively, as we believe that it was effective in promoting good scar maturation in the small areas with skin defects in our case.

## Conclusions

Colchicine is frequently used in Behçet's disease. These patients can be operated without any problems by continuing to use low-dose colchicine. Low-dose colchicine has a positive effect on wound healing by reducing inflammation in the early period and providing scar maturation in the late period.
